# UNR/*CDSE1* expression as prognosis biomarker in resectable pancreatic ductal adenocarcinoma patients: A proof-of-concept

**DOI:** 10.1371/journal.pone.0182044

**Published:** 2017-08-01

**Authors:** Javier Martinez-Useros, Tihomir Georgiev-Hristov, María Jesús Fernández-Aceñero, Aurea Borrero-Palacios, Alberto Indacochea, Santiago Guerrero, Weiyao Li, Arancha Cebrián, Teresa Gómez del Pulgar, Alberto Puime-Otin, Laura del Puerto-Nevado, María Rodríguez-Remírez, Nuria Pérez, Angel Celdrán, Fátima Gebauer, Jesus Garcia-Foncillas

**Affiliations:** 1 Translational Oncology Division, OncoHealth Institute, University Hospital Fundacion Jimenez Diaz (FJD), Madrid, Spain; 2 Surgery Department, Hospital de Villalba, Collado-Villalba, Spain; 3 Department of Pathology, Clinico San Carlos University Hospital, C/ Profesor Martin Lagos, Madrid, Spain; 4 Gene Regulation, Stem Cells and Cancer Programme, Centre for Genomic Regulation (CRG), The Barcelona Institute of Science and Technology, Barcelona, Spain; 5 Oncology and Molecular Pathology Research Group-VHIR- Vall d' Hebron Institut de Recerca-Vall d' Hebron Hospital, P/ de la Vall d'Hebron, Barcelona, Spain; 6 Universitat Pompeu Fabra (UPF), Barcelona, Spain; 7 Department of Pathology, University Hospital Fundacion Jimenez Diaz, Madrid, Spain; 8 Hepatobiliary and Pancreatic Surgery Unit, General and Digestive Tract Surgery Department, Fundacion Jimenez Diaz University Hospital, Madrid, Spain; Mayo Clinic Rochester, UNITED STATES

## Abstract

Pancreatic ductal adenocarcinoma is an aggressive form of pancreatic cancer and the fourth leading cause of cancer-related death. When possible, curative approaches are based on surgical resection, though not every patient is a candidate for surgery. There are clinical guidelines for the management of these patients that offer different treatment options depending on the clinical and pathologic characteristics. However, the survival rates seen in this kind of patients are still low. The *CDSE1* gene is located upstream of *NRAS* and encodes an RNA-binding protein termed UNR. The aim of this study was to analyze UNR expression and its correlation with outcome in patients with resectable pancreatic ductal adenocarcinoma (PDAC). For this, samples from resectable PDAC patients who underwent duodenopancreatectomy were used to evaluate UNR protein expression by immunohistochemistry using a tissue microarray. Here, we observed that low UNR expression was significantly associated with shorter progression-free survival after surgery (*P =* 0.010). Moreover, this prognostic marker remained significant after Cox proportional hazards model (*P =* 0.036). We further studied the role of *CDSE1* expression in patient’s prognosis using data from public repositories (GEO and TGCA), confirming our results. Interestingly, *CDSE1* expression correlated with that of genes characteristic of an immunogenic molecular subtype of pancreatic cancer. Based on these findings, UNR may be considered a potential prognostic biomarker for resectable PDAC and may serve to guide subsequent adjuvant treatment decisions.

## Introduction

Pancreatic ductal adenocarcinoma (PDAC) has higher incidence in industrialised countries [1] and is the fourth leading cause of cancer death in both sexes in the USA, where 53,070 new cases of PDAC were diagnosed in 2016 [2]. Moreover, it is the eighth leading cause of cancer death in men and the nineth in women worldwide [3]. It has been reported that the 5-year survival rate is 50% when tumors are < 2 cm in size [4] and close to 100% for tumors < 1 cm [5]. Although these data are encouraging, PDAC is usually asymptomatic, and the disease only becomes apparent after the tumor invades surrounding tissues or metastasizes to distant organs [6]. In fact, distant metastasis is found in 53% of PDAC patients at the time of diagnosis [2]. To date, surgical resection remains the best management option for PDAC originating in the ampulla of Vater, bile duct, or pancreas. Patient’s prognosis has been predicted based on pathological characteristics such as tumor size, grade of differentiation, lymph-node status, etc [7]. Several prognostic biomarkers have been suggested, such as Smad4 or MUC1; also, predictive biomarkers including SPARC, HuR, or members of the BRCA2 family have been described [8–11]. To date, preoperative levels of carbohydrate antigen 19–9 (CA 19–9) are the only prognostic biomarker approved by the Food and Drug Administration (FDA) for use in cases of resectable PDAC [12]. This marker shows a relatively high sensitivity and specificity for PDAC [13], providing results that are superior to those of other markers, such as carcinoembryonic antigen (CEA), carbohydrate antigen 50 (CA-50), and DUPAN-2 [14, 15]. However, the applicability of CA 19–9 is compromised by the fact that biliary obstruction can increase its serum levels [16], and up to 10% of the population cannot synthesise this antigen [17].

In the late 1980s, an active transcription unit called UNR (Upstream of N-ras) was discovered and subsequently included in the RNA-binding protein (RBP) family due to its ability to bind single-stranded RNA [18]. RBPs are pivotal components in the determination of messenger RNA (mRNA) and microRNA function, as they control transcript biogenesis, localization, degradation, and activity. Alteration of RBP function can lead to impairment of any of the crucial steps of RNA processing, and deregulation of RBP expression or activity has been reported in several malignancies [19]. Moreover, several RBPs have been shown to play a key role in cancer via regulation of mRNA splicing, translation, and stability [20]. *In vitro* assays indicated that UNR could interact with cytoplasmic RNA in a sequence-specific manner [18, 21]. Subsequent studies demonstrated that UNR acts as an RNA chaperone by changing the structure of the IRES into one that is functionally competent for translation [22]. Other reports showed that UNR compensates X-chromosome dosage in Drosophila [23] and prevents differentiation of embryonic stem cells in mouse models [24].

In the cancer context, UNR has been shown to regulate proto-oncogenes like c-fos [25] and c-myc [26]. In addition, UNR promotes melanoma progression by regulating the expression of Pten, Rac1 and Vimentin, among other genes [27]. Interestingly, overexpression of *HEPSIN*, one of the most consistently up-regulated genes in prostate-cancer patients [28], inhibits the expression and IRES activity of UNR in cancer-derived cell lines [29]. In contrast, knock-down of *HEPSIN* expression with siRNA led to an increase of UNR and up-regulation of its IRES activity [29]. Curiously, UNR is transcribed from the same strand of DNA as the *NRAS* proto-oncogene [30], and its expression has been reported to down-modulate *NRAS* expression through mRNA accumulation in tissues [31]. Altogether, these data point to diverse roles of UNR in cancer development.

The role of UNR in PDAC has not been previously addressed. In this study, we aimed to quantify UNR protein expression and evaluate its role as a potential marker to determine outcome of PDAC patients. We have further analysed the association between UNR/*CDSE1* expression and different molecular subtypes of pancreatic cancer.

## Materials and methods

### Patient samples

A total of 53 patients with pancreatic adenocarcinoma who underwent pancreaticoduonenectomy from 2007 to 2013 at the Hepatobiliary and Pancreatic Surgery Unit (General and Digestive Tract Surgery Department, Fundación Jiménez Díaz University Hospital) were assessed for eligibility. All cephalic duodenopancreatectomy specimens have been sectioned and embedded *in toto* following Verbeke *et al*. scheme [32]. This scheme allows accurate establishment of the origin of the tumor in the pancreas, the extrahepatic biliary tract or the duodenum. Twenty-two patients were excluded due to insufficient sample quality for immunohistochemistry, patients lost to follow-up, or tumors having duodenal origin. Most of the tumors studied were in stage II (78%). Gemcitabine was administered alone or in combination with radiotherapy as adjuvant treatment post-surgery in one-third of the cases included (32%). All tumor samples included in this study were confirmed to be low-grade resectable pancreatic adenocarcinomas based on the recommendations of the College of American Pathologists [33].

### Immunohistochemistry and quantification

A tissue microarray was constructed for immunohistochemistry analysis and contained 62 cores (2 cores per patient) using the MTA-1 tissue arrayer (Beecher Instruments, Sun Prairie, USA). Each core (diameter, 1 mm) was punched from pre-selected tumor regions in paraffin-embedded tissues. Staining was conducted in 2-μm sections. Slides were deparaffinised by incubation at 60°C for 10 min and incubated with PT-Link (Dako, Denmark) for 20 min at 95°C in a high pH buffered solution. To block endogenous peroxidase, holders were incubated with peroxidase blocking reagent (Dako, Denmark). Biopsies were incubated for 20 min with a 1:50 dilution of CDSE1 antibody (ab96124; Abcam, Cambridge, UK) and 1:1000 dilution of NRAS antibody (ab167136; Abcam, Cambridge, UK) followed by incubation with the appropriate anti-Ig horseradish peroxidase-conjugated polymer (EnVision, Dako, Denmark) to detect antigen-antibody reaction. Both CDSE1 antibody and anti-Ig horseradish peroxidase-conjugated antibody presented high specificity and no positiveness resulted from these antibodies individually. A human intestinal tissue was used as a positive control (according to the human protein atlas available at http://www.proteinatlas.org) for immunohistochemical staining and to determine CDSE1 antibody concentration. Sections were then visualised with 3,3’-diaminobenzidine as a chromogen for 5 min and counterstained with haematoxylin. Photographs were taken with a stereo microscope (Leica DMi1, Wetzlar, Germany). Immunoreactivity of tumor sample was quantified blind with UNR intensity of expression categorized as negative, low, medium or high expression according to Wurth *et al*. [27]. Quantification for each patient biopsy was calculated with the average of both cores by two independent pathologists.

### Statistical analysis of immunohistochemical expression

The association between UNR expression and progression-free survival after resection was the primary endpoint, and overall survival was the secondary endpoint. Progression-free survival was defined as the interval between the dates of surgery and recurrence (local or distant). Overall survival was defined as the interval between the dates of surgery and death from any cause.

The association between UNR expression and clinico-pathological variables was evaluated by Fisher´s exact test.

The univariate Cox proportional hazards model was used to assess the hazard ratios and confidence intervals of both molecular and clinical variables.

### TCGA-pancreatic cancer dataset analysis

Sixty patients from a group of 186 pancreatic cancer patients with RNA expression data in the TCGA database were eligible for overall survival analysis, while 47 patients were eligible for progression-free survival analysis (S1 Fig). We selected stages I/II low grade PDAC patients featuring histology with complete resections (R0) and follow-up, without *CDSE1* genetic alterations and untreated with neoadjuvant chemotherapy. For both progression-free and overall survival, ROC (Receiver Operating Characteristic) curves did not show a clear cut-off point (progression-free survival AUC = 0.578, *P =* 0.129; overall survival AUC = 0.583, *P =* 0.065; data not shown). Therefore, mean of Z-score was used as cut-off point for both survival analyses. Additionally, the TCGA dataset was analysed using cBioPortal [34, 35] to address gene expression and to calculate Pearson and Spearman correlation coefficients. Correlation coefficients were interpreted according to Cohen [36]. Values of 0.10 to 0.30 could be interpreted as a weak correlation, 0.30 to 0.50 as a moderate correlation and greater than 0.50 as a strong correlation [36]. Z-scores were plotted in a heatmap using Perseus_1.5.3.0.

### GEO (GSE28735) dataset analysis

Survival analysis was assessed with the association between *CDSE1* Z-score and overall survival information of 42 pancreatic tumors that contained complete clinical follow-up from Gene Expression Omnibus (GEO; http://www.ncbi.nlm.nih.gov/geo) dataset with accession number GSE28735 entitled: “Microarray gene-expression profiles of 45 matching pairs of pancreatic tumor and adjacent non-tumor tissues from 45 patients with pancreatic ductal adenocarcinoma”. Expression profile of tumor samples were detected with Affymetrix GeneChip Human Gene 1.0 ST arrays. Z-score was stratified into tertiles (low ≤ 33%; 34% < medium ≤ 66%; high > 67%), and third tertile (high expression) was used as cut-off point.

Z-score for *CDSE1* mRNA expression was calculated as follows: Z-score = (log value of mRNA expression in tumor sample–log value of mRNA mean expression in reference samples) / log value of standard deviation of mRNA expression in reference samples. Reference samples have been considered the adjacent non-tumor tissues (for GSE28735 dataset) and all diploid tumors for the gene in question (for TCGA dataset). All survival curves were generated using the Kaplan-Meier method, and significant differences in survival between groups were determined by the log-rank test. P-values ≤ 0.05 were considered significant. Analysis was performed with the IBM SPSS programme, version 20.0.

## Results

### Patient characteristics

The clinical features of the PDAC patients included in the study are summarised in Table 1. Our cohort was well-balanced in terms of sex (48% males and 52% females). The median age of patients was 69 years (range 37–82 years). Pathologic diagnosis revealed the size of the resected tumors to be lower than 2 cm in 61% of cases. Twenty-two percent of tumors were stage I and 78% stage II. Negative surgical margins were found after surgery in 90% of cases. Fifty-eight percent of patients showed lymph-node involvement and most patients had neural and vascular invasion (74% and 71%, respectively). Adjuvant treatment based on gemcitabine alone or gemcitabine plus radiotherapy was administered post-surgery in 32% of patients based on the consensus of a multidisciplinary team. Gemcitabine was administered in 3–12 cycles depending on radiotherapy doses (45–54 Gy in 1.8–2.5 Gy fractions).

**Table 1:** Clinical characteristics of resectable low-grade pancreatic cancer patients.

Characteristics	N (%)
**Age**	
< 65 years	12 (39%)
> 65 years	19 (61%)
**Sex**	
Female	16 (52%)
Male	15 (48%)
**Size**	
< 2 cm	19 (61%)
> 2 cm	12 (39%)
**Stage**	
I	7 (23%)
II	24 (77%)
**pT**	
T1	5 (16%)
T2	3 (10%)
T3	23 (74%)
**pN**	
N0	12 (39%)
N1	18 (58%)
N/A	1 (3%)
**Tumor location**	
Pancreas	12 (39%)
Bile duct	10 (32%)
Ampulla	9 (29%)
**Lymph nodes involved**	
No	12 (39%)
Yes	18 (58%)
N/A	1 (3%)
**Adjuvant treatment**	
No	20 (65%)
Yes	10 (32%)
N/A	1 (3%)
**Positive margins**	
No	28 (90%)
Yes	3 (10%)
**Vascular invasion**	
No	9 (29%)
Yes	22 (71%)
**Neural invasion**	
No	8 (26%)
Yes	23 (74%)

N/A: not available

### Low UNR expression level is associated with poor outcome in low-grade resected PDAC patients

To date, outcome of resected PDAC patients is clinically predicted according to pathologic criteria. For this reason, we first checked the statistical power of stage as a prognostic tool in our cohort of patients. For that purpose, the association between stage and survival of PDAC patients was assessed. However, Kaplan-Meier analysis revealed no statistically significant association between stage and progression-free survival (*P =* 0.196; data not shown) nor with overall survival (*P =* 0.657; data not shown).

**Fig 1:**
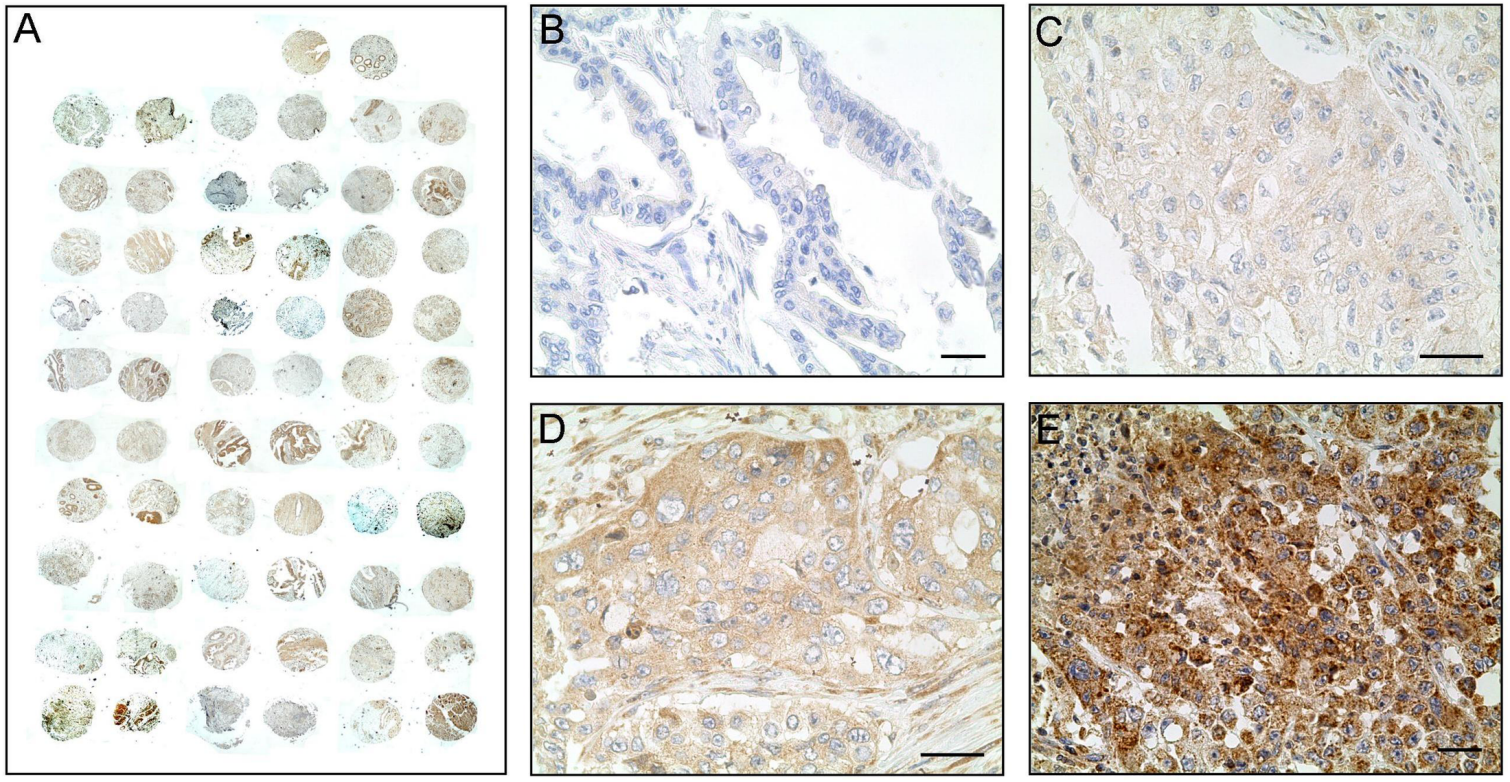
UNR immunostaining. A) The TMA slide contained 62 tumor tissue cores (2 cores per patient) and was immunostained with the anti-CSDE1 antibody. Representative images of tumor samples exhibiting negative UNR expression (B), low (C), medium (D) and high UNR expression (E). Scale bar: 10 μm.

Based on previous reports suggesting an association between RBPs and cancer, we hypothesised that UNR expression levels could be closely related to outcome in patients with PDAC. To test this hypothesis, a tissue microarray was constructed and stained to quantify UNR expression (Fig 1A). We stratified pancreatic cancer samples with differential UNR expression from negative to highly positive (Fig 1B–1E). All samples that stained positive exhibited a cytoplasmic expression pattern and some diffuse membrane localisation (Fig 1C–1E).

**Fig 2:**
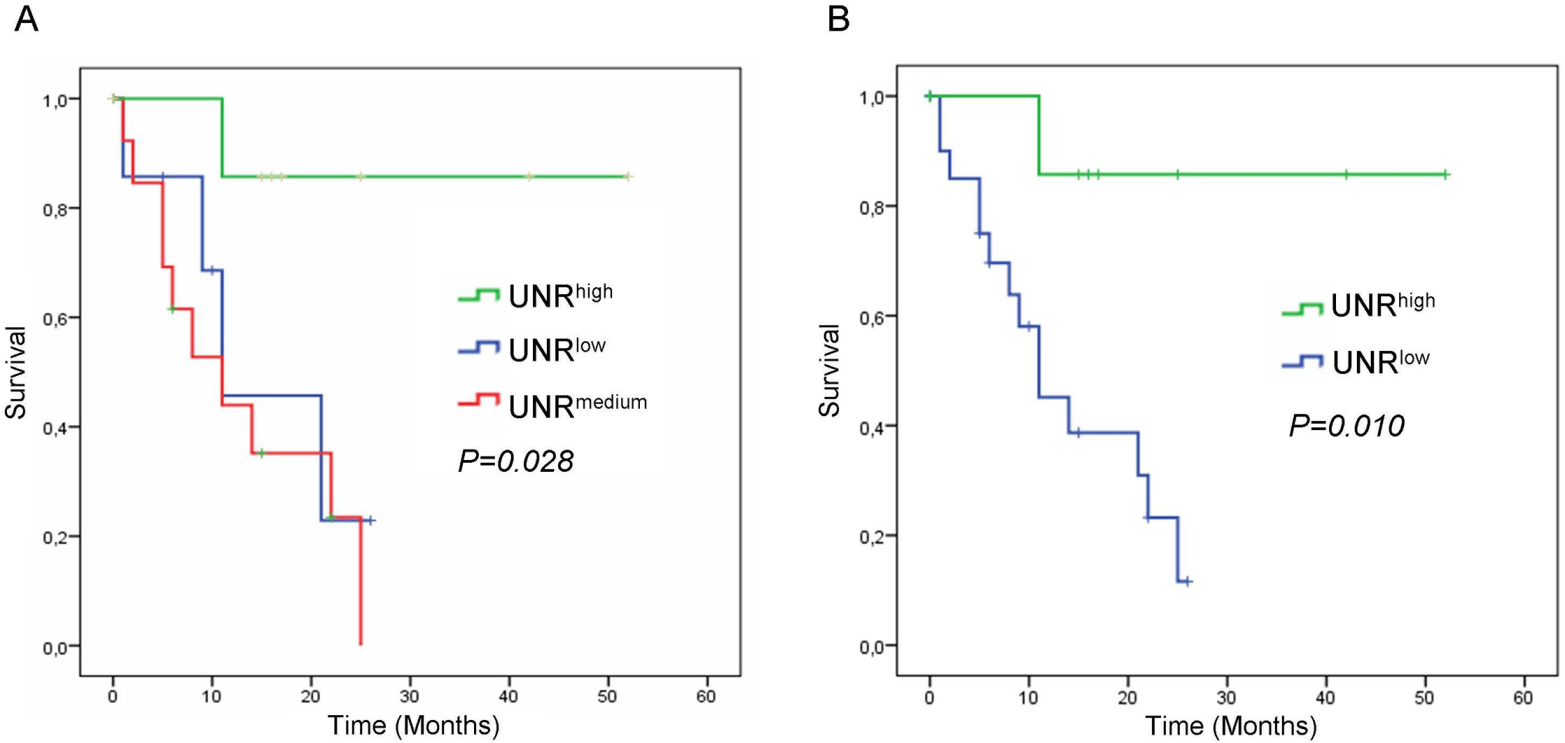
Kaplan-Meier analysis for progression-free survival after surgery based on UNR expression levels in low-grade resectable PDAC patients. A) Survival curves according to UNR expression stratified in tertiles. B) Survival curves of PDAC patients according to low or high UNR expression.

Subsequently, the association between UNR expression and outcome was assessed. Interestingly, it was observed that patients with negative/low or medium expression had similar behaviour according to progression-free survival, while patients with high expression clearly presented a better outcome (*P =* 0.028; Fig 2A). Therefore, high expression was established as cut-off point yielding two groups, with high- and low-risk according to low or high UNR expression, respectively.

Survival analysis performed with low or high expression of UNR showed shorter progression-free survival in the arm with low UNR expression (*P =* 0.010) (Fig 2B). Mean progression-free survival for patients expressing low levels of UNR was 13 months (range 9–17 months), while mean survival for those expressing high levels of UNR was 46 months (35–56 months) (Table 2). Median revealed that patients with low levels of UNR took 11 months to experience disease recurrence (range 5–17 months), while the median was not reached in the case of patients with high UNR levels (Table 2).

**Table 2:** Progression-free survival (months) according to UNR expression.

		Mean			Median		
		95%	CI		95%	CI	
UNR	Months	Lower	Upper	Months	Lower	Upper	*P-value*
Low	13.576	9.453	17.700	11.000	4.925	17.075	0.010
High	46.143	35.514	56.771	-	-	-	

CI: confidence interval

In order to compare the potential prognosis value of UNR expression with the other clinical variables we performed a Cox proportional hazards model. The univariate analysis for progression-free survival confirmed that patients with low expression of UNR showed higher risk of recurrence after surgery compared to those with high expression of UNR (HR = 8.914; *P =* 0.036) (Table 3). Moreover, UNR expression remained the only significant variable in this analysis.

**Table 3:** The effect of the molecular and clinical variables on progression-free survival in resectable low-grade pancreatic cancer patients.

		Univariate		
		95%	CI	
	HR	Lower	Upper	*P-value*
**Age**				0.588
> 65 years *vs* < 65 years	1.313	0.490	3.518	
**Sex**				0.540
Male *vs* Female	1.336	0.528	3.381	
**Adjuvant treatment**				0.329
No *vs* Yes	1.718	0.579	5.093	
**Tumor size**				0.926
>2 cm *vs* <2cm	1.050	0.373	2.959	
**Stage**				0.173
II *vs* I	2.540	0.571	11.306	
**pT**				0.341
T3 *vs* T1-T2	1.854	0.521	6.601	
**pN**				0.565
N1 *vs* N0	1.385	0.461	4.159	
**Tumor location**				0.263
Pancreas *vs* Others	1.924	0.611	6.053	
**Vascular Invasion**				0.728
Yes *vs* No	1.220	0.399	3.731	
**Neural Invasion**				0.728
Yes *vs* No	1.220	0.399	3.731	
**Lymph nodes affected**				0.312
Yes *vs* No	1.719	0.602	4.911	
**UNR**				0.036
Low *vs* High	8.914	1.159	68.584	

HR: hazard ratio; CI: confidence interval; *vs*: versus

Overall survival was analysed as a secondary endpoint. However, we did not find any statistically significant difference between arms with high or low UNR expression levels (*P =* 0.429; data not shown).

To verify if expression of UNR/*CDSE1* could be related to any clinico-pathological variable a crosstab was performed thereafter (Table 4). Here, there were no statistically significant associations between UNR expression and all variables of the study. This analysis included gender (*P =* 0.704), age (*P =* 1.000), stage (*P =* 0.150), pT (*P =* 0.185), pN (*P =* 0.418), tumor size (*P =* 1.000), lymph-node involvement (*P =* 0.418), neural invasion (*P =* 0.185) and positive margins of resection (*P =* 1.000). Interestingly, low UNR expression showed a high trend towards significance with vascular invasion (*P =* 0.077) (Table 4).

**Table 4:** Association between UNR expression and clinico-pathological parameters.

	UNR^low^	UNR^high^	
Parameters	N	N	*P-value*
**Gender**			0.704
Female	12	4	
Male	10	5	
**Age**			1.000
< 65 years	9	3	
> 65 years	13	6	
**Stage**			0.150
I	3	4	
II	19	5	
**pT**			0.185
T1-T2	4	4	
T3	18	5	
**pN**			0.418
N0	7	5	
N1	14	4	
**Size**			1.000
< 2 cm	13	6	
> 2 cm	9	3	
**Lymph nodes involved**			0.418
No	7	5	
Yes	14	4	
**Vascular Invasion**			0.077
No	4	5	
Yes	18	4	
**Neural Invasion**			0.185
No	4	4	
Yes	18	5	
**Positive margins**			1.000
No	20	8	
Yes	2	1	

N: number of patients

Since the *CDSE1* locus is only 150 nucleotides upstream of the *NRAS* gene and its regulation has been previously correlated with UNR expression [30], NRAS protein was also quantified by immunohistochemistry and a link between UNR/*CDSE1* and *NRAS* expression was evaluated. Nevertheless, no correlation was found between the expression levels of both proteins (*P =* 0.903). Additionally, a survival analysis performed with Kaplan-Meier plots confirmed the lack of association; instead, a high trend towards significance was found between *NRAS* expression and both progression-free survival (*P =* 0.054) and overall survival (*P =* 0.092) in this set of patients (data not shown).

### Survival analysis according to UNR/*CSDE1* expression in PDAC validation cohorts

We next analysed survival according *CDSE1 mRNA* expression on two independent datasets of pancreatic cancer patients used as validation sets. One cohort was taken from The Cancer Genome Atlas (TCGA) using the cBioPortal Interface [34, 35], and the other was taken from Gene Expression Omnibus (GEO) database.

Patients from TGCA that presented non-cancer related death, incomplete resections (R1), neuroendocrine origin, high-grade of differentiation, stage III/IV, *CDSE1* mutations, treated with neoadjuvant chemotherapy, or missed *CDSE1* expression data or clinical/pathological information where excluded from the study (S1A Fig). Progression-free survival analysis of 47 eligible patients showed that patients with high *CDSE1* expression presented better survival compared to low *CDSE1* expression cases (*P =* 0.009; median survival of 28 months vs. 14 months, respectively) (S1B Fig). Overall survival analysis with 60 patients did not achieve statistical significance; however, a high trend toward significance was found between patients with high and low *CDSE1* expression (*P =* 0.056). Here, patients with high *CDSE1* expression presented longer overall survival (median survival of 30 months, compared to 20 months for patients with low CSDE1 expression) (S1C Fig).

All patients from GEO database were included in the study except for those with no survival information (n = 3). As this dataset lacks information on pathology, we included all patients with no inclusion/exclusion criteria. Perhaps not surprisingly, given that patients were analysed independently of grade of differentiation, stage, treatment or positive resection margins, overall survival analysis revealed no statistical significance between high or low *CDSE1* expression (*P =* 0.129). However, patients with high *CDSE1* expression showed longer median overall survival than patients with low *CDSE1* expression (median overall survival 21 months vs. 13 months, respectively) (S2 Fig). Altogether, the results from both validation sets support the observation that high UNR/*CDSE1* expression correlates with better outcome in resectable PDAC patients.

### The expression of *CDSE1* is associated to the immunogenic molecular subtype of pancreatic cancer

**Fig 3:**
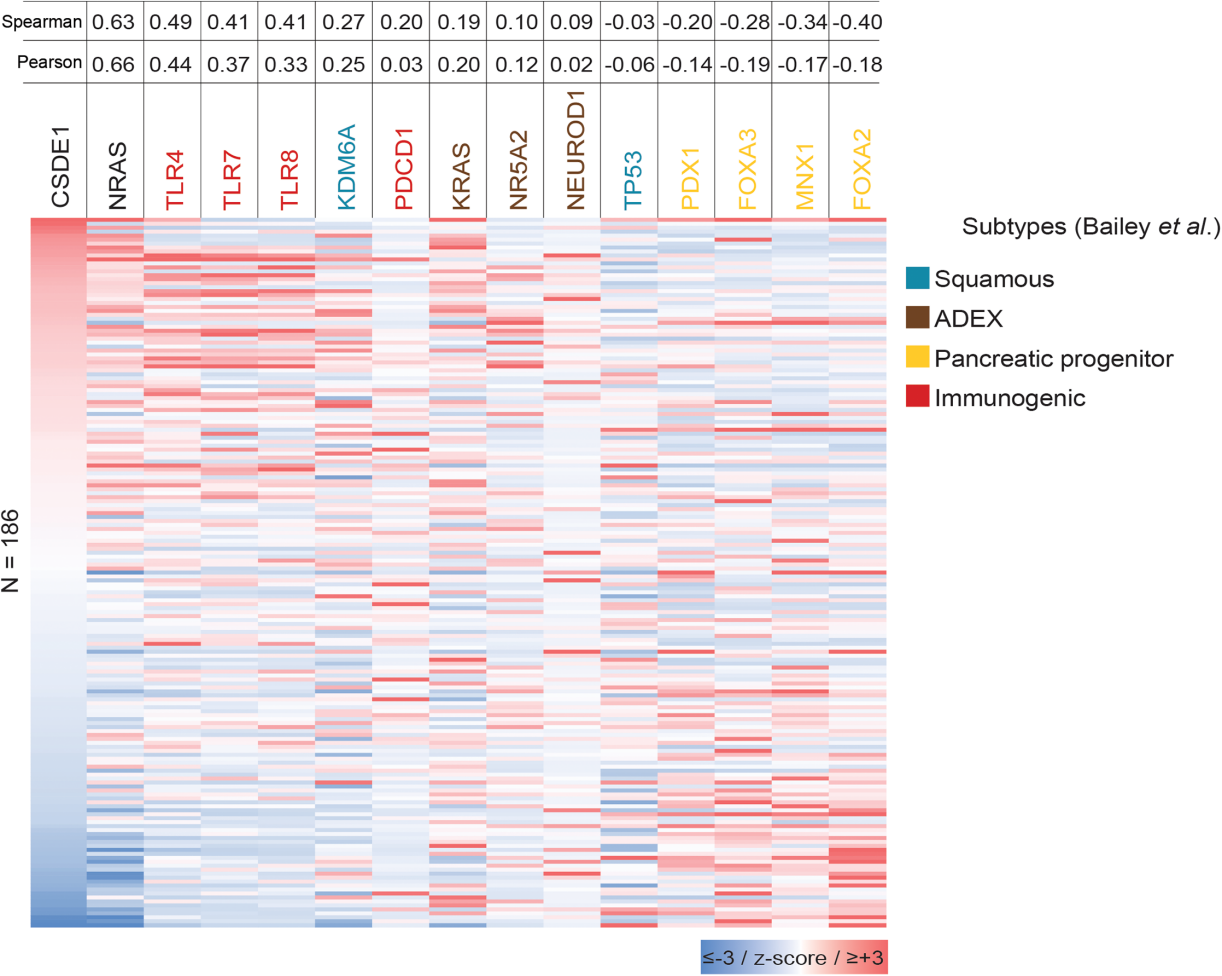
Heatmap comparison of Z-scores that correlated with *CSDE1* expression. Spearman and Pearson analyses show correlation between *CSDE1* expression and the main genes of Bailey´s molecular subtypes of pancreatic cancer.

The mRNA expression profile of 186 pancreatic cancer patients from the TGCA dataset was correlated with the expression of *CSDE1* using Spearman and Pearson tests. Here, the expression of *CDSE1* and *NRAS* transcripts correlated (Spearman = 0.63; Pearson = 0.66) (Fig 3). Interestingly, we found a moderate correlation between *CDSE1* and *TLR4* (Spearman = 0.49; Pearson = 0.44), *TLR7* (Spearman = 0.41; Pearson = 0.37), and *TLR8* expression (Spearman = 0.41; Pearson = 0.33) (Fig 3). The expression of these Toll-like receptor genes has been associated with the pancreatic cancer immunogenic subtype defined by Bailey *et al*. [37]. It was reported that patients classified under the immunogenic subtype present a better prognosis compared to the other subtypes: ADEX (abnormally differentiated endocrine exocrine), progenitor and squamous subtype (median survival of 30.0, 23.7, 25.6 and 13.3 months, respectively) [37]. On the other hand, *CDSE1* expression showed negative correlation with progenitor subtype genes such as *PDX1* (Spearman = -0.20; Pearson = -0.14), *FOXA3* (Spearman = -0.28; Pearson = -0.19), *MNX1* (Spearman = -0.34; Pearson = -0.17) and *FOXA2* (Spearman = -0.40; Pearson = -0.18) (Fig 3).

Overall, consistent with our immunohistochemistry data, these *in silico* analyses support the notion that UNR/*CDSE1* expression predicts better outcome in resectable PDAC patients. Further analyses using larger patient cohorts should be performed to confirm these promising pilot results.

## Discussion

PDAC is rare, although due to its poor clinical outcome it is the fourth leading cause of cancer death. A demographic report showed that the incidence of this cancer is rising worldwide [2], possibly associated with an increase in consumption of sugar, high-carbohydrate-content foods, red and processed meat or obesity [38–40]. The most effective standard treatment consists of pancreatectomy performed by Whipple procedure [41]. Oncology guidelines are useful to manage this kind of patients [42, 43]. Although treatment options for this cancer are increasing [44–46], mortality continues around 74% within the first year of diagnosis. It is therefore imperative to find new treatments, predictive tools and translational prognostic biomarkers to personalise the therapy and improve survival [47].

Post-transcriptional gene regulation is a rapid and efficient way to adjust the proteome of a cell to environments in constant variation. RBPs regulate post-transcriptional gene expression during biological processes such as cell proliferation, differentiation, invasion, metastasis, and apoptosis [20]. In addition, RBPs bind hundreds of mRNAs to form complex networks that are crucial for tumor development. UNR is an RBP related with multiple processes, such as apoptosis [48], stem-cell differentiation [24] and the migration of pre-cerebellar neurons [49]. Regarding cancer, UNR has been considered a pro-oncogenic factor for its role in stabilising c-fos mRNA and simulating the translation of c-myc mRNA [25, 26], and promoting melanoma metastasis [27]. However, upregulation of UNR is not always associated to tumor progression, indicating that the precise role of UNR in cancer depends on context. For example, overexpression of the *HEPSIN* oncogene in prostate cancer [28] downregulates the expression and IRES activity of UNR [29]. Consistent with a protective effect of UNR, we describe here an association between low levels of UNR and poor clinical outcome of PDAC patients. It has been described an association between *CSDE1* mRNA and protein expression along cell cycle [50, 51]. Thus, we analysed two independent datasets based on mRNA expression profile, and *CSDE1* expression results were in agreement with our previous findings. These results are in line with those of Cornelis *et al*. reporting that a constitutive high expression of UNR becomes cytotoxic and leads to cell death [52]. In the same vein, UNR-deficient murine embryonic stem cells display resistance to apoptosis after irradiation [48]. Thus, in certain cancer types UNR may act to suppress tumor formation.

The available expression profile of 186 pancreatic cancer patients from TGCA database allowed us to correlate *CDSE1* expression to genes associated with specific molecular subtypes of pancreatic cancer. In this analysis, *CDSE1* presented a moderate correlation with genes involved in Toll-like receptor signalling pathway. This pathway mediates innate immunity and triggers pro-inflammatory signalling cascades [53]. The correlation between *CDSE1* and *TLR4*, *TLR7* or *TLR8* expression suggests that PDAC patients with high UNR/*CDSE1* expression may present a less aggressive tumor phenotype, more susceptible to be cleared by the immune response [54, 55].

The *CDSE1* and *NRAS* loci are located close together in the genome, with an intergenic distance of only 150 nucleotides. This special location raised the possibility of transcriptional interference between both genes. Indeed, such interference was found in mouse tissues, where deletion of the *CSDE1* promoter led to an increase in *NRAS* mRNA accumulation [29]. Contrary to results in the mouse, however, we find no evidence for an anti-correlation in human tumor samples. Rather, we find a direct correlation between *CSDE1* and *NRAS* mRNA levels in PDAC samples from the TGCA database. Furthermore, this correlation is not maintained at the protein level, as we found no relationship between CSDE1 and NRAS protein levels by immunohistochemistry. Therefore, the protective role of CSDE1 is not explained by simple down-regulation of NRAS, and must rely on other targets.

Future experiments should be directed towards the identification of these targets. In the meantime, our results provide a proof-of-concept study supporting UNR/*CDSE1* expression as a potential biomarker for PDAC prognosis.

## Conclusions

Here, we describe the association between low UNR expression and poor outcome of low-grade resectable PDAC patients. Low expression of UNR showed a statistical trend when it was associated with vascular invasion and other clinico-pathological characteristics like neural invasion, pT and stage, indicating UNR loss as a feasible factor to induce malignant phenotype, and therefore, a poor outcome event in PDAC development. Furthermore, UNR expression was associated with immunogenic phenotype of pancreatic cancer. Based on these findings, we propose UNR/CSDE1 as an independent prognostic biomarker for resectable pancreatic cancer.

## Supporting information

S1 Fig Survival analysis of TGCA validation set according *CSDE1* expression. A) Flow chart of the selected population and exclusion criteria. B) Kaplan–Meier analysis for progression-free survival and overall survival (C) based on *CSDE1* mRNA expression level.
(TIF)

S2 Fig Kaplan-Meier analysis of overall survival of GEO validation set (GSE28735) according *CSDE1* expression. (TIF)

S1 Table Clinical and pathological information of patients recruited in the study. (DOC)
