# Supplementary figures and images for: UNR/CDSE1 expression as prognosis biomarker in resectable pancreatic ductal adenocarcinoma patients: A proof-of-concept
Source: PLoS One. 2017 Aug 1;12(8):e0182044. doi: 10.1371/journal.pone.0182044 (PMC5538752; doi:10.1371/journal.pone.0182044)

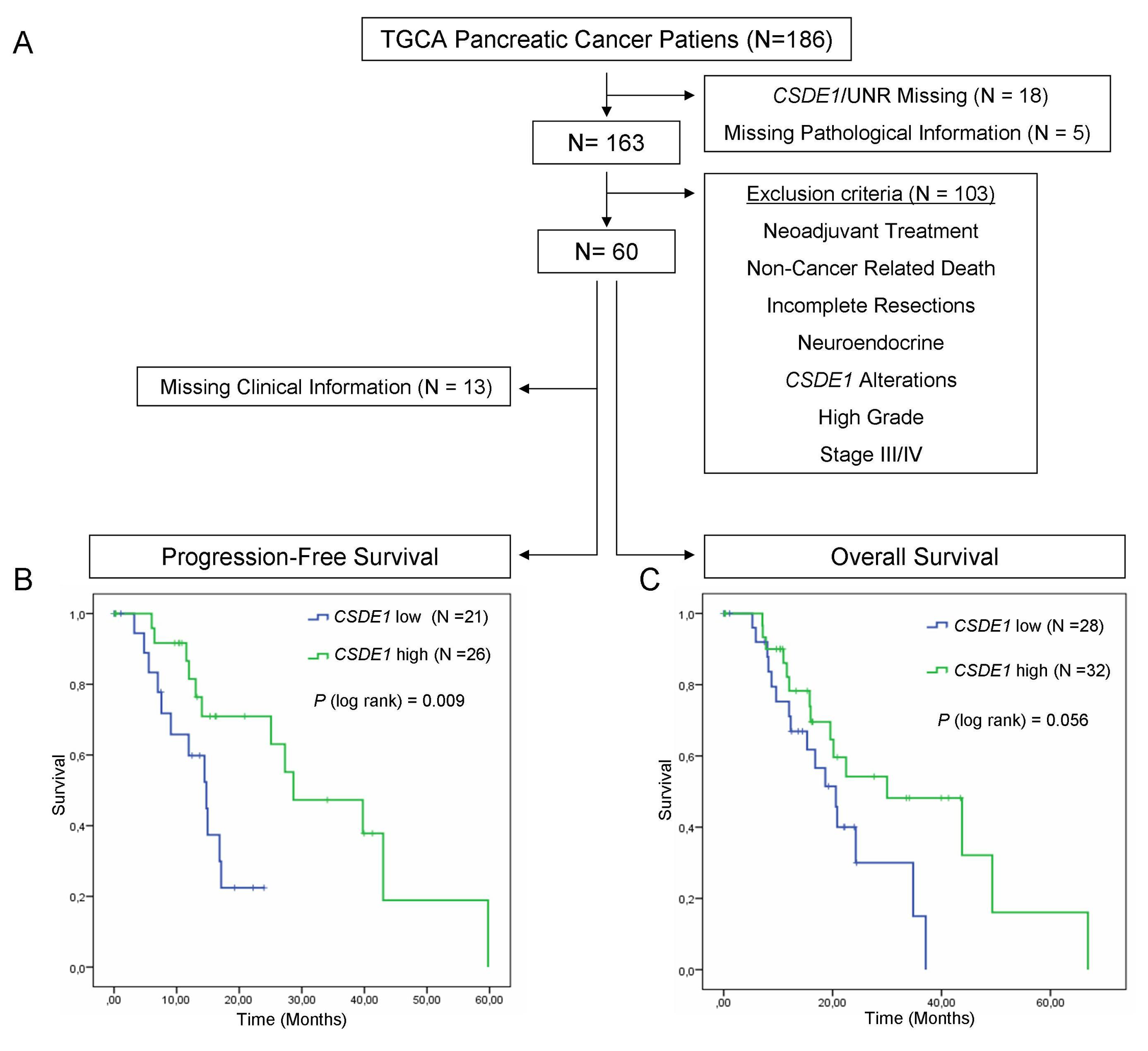

Supplement: S1 Fig — A) Flow chart of the selected population and exclusion criteria. B) Kaplan–Meier analysis for progression-free survival and overall survival (C) based on CSDE1 mRNA expression level. (TIF) [file pone.0182044.s001.tif]

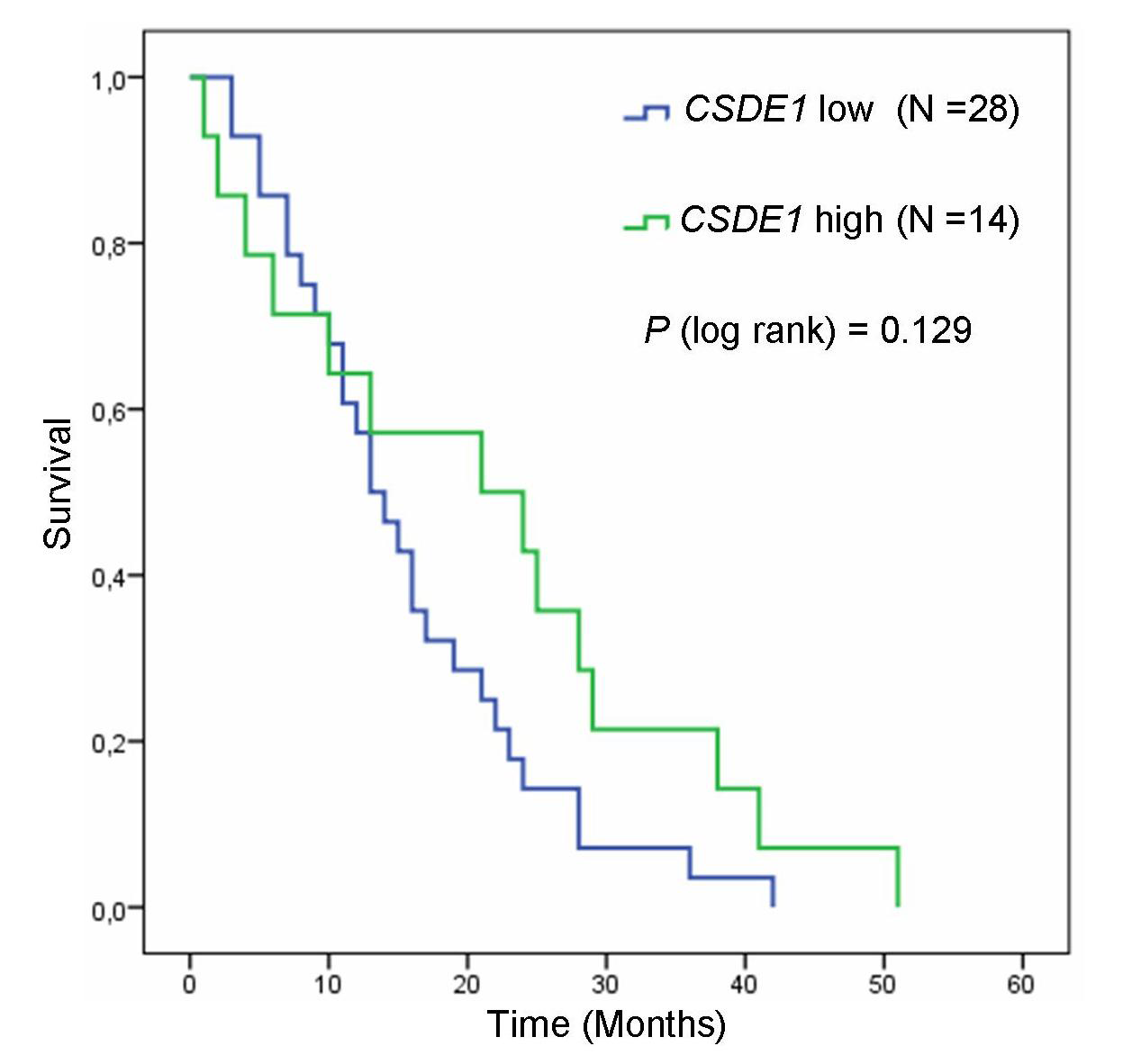

Supplement: S2 Fig — (TIF) [file pone.0182044.s002.tif]
