# Supplementary material for: UNR/CDSE1 expression as prognosis biomarker in resectable pancreatic ductal adenocarcinoma patients: A proof-of-concept
Source: PLoS One. 2017 Aug 1;12(8):e0182044. doi: 10.1371/journal.pone.0182044 (PMC5538752; doi:10.1371/journal.pone.0182044)
Supplement: S1 Table — (DOC) [file pone.0182044.s003.doc]

| Sample | Age | Gender | Tumor size | Stage | pTN | Origin | Adjuvant treatment | R | Vascular invasion | Neural invasion | UNR expression | PFS (months) | Progression Event | OS (months) | Survival event |
| --- | --- | --- | --- | --- | --- | --- | --- | --- | --- | --- | --- | --- | --- | --- | --- |
| 1 | 64 | Male | < 2 cm | 1A | T1N0 | Ampulla | None | R0 | No | No | High | 0 | No | 0 | Dead |
| 2 | 70 | Female | < 2 cm | 1A | T1N0 | Ampulla | None | R0 | No | No | High | 11 | Yes | 36 | Alive |
| 3 | 73 | Male | > 2 cm | 2B | T3N1 | Biliar duct | Gemcitabine | R0 | Yes | Yes | High | 15 | No | 20 | Alive |
| 4 | 77 | Male | < 2 cm | 2A | T3N0 | Biliar duct | None | R1 | No | Yes | High | 0 | No | 0 | Alive |
| 5 | 79 | Male | < 2 cm | 1A | T1N0 | Pancreas | None | R0 | Yes | Yes | High | 52 | No | 52 | Alive |
| 6 | 60 | Female | > 2 cm | 2B | T3N1 | Ampulla | Gem + RT | R0 | Yes | Yes | High | 42 | No | 42 | Alive |
| 7 | 54 | Female | < 2 cm | 2B | T3N1 | Biliar duct | Gemcitabine | R0 | No | No | High | 25 | No | 25 | Alive |
| 8 | 69 | Male | > 2 cm | 2B | T3N1 | Biliar duct | None | R0 | Yes | Yes | High | 17 | No | 17 | Alive |
| 9 | 77 | Female | < 2 cm | 1A | T1N0 | Pancreas | None | R0 | No | No | High | 16 | No | 16 | Alive |
| 10 | 65 | Male | < 2 cm | 1A | T1N0 | Pancreas | None | R0 | No | No | Medium | 6 | No | 6 | Alive |
| 11 | 37 | Male | > 2 cm | 2B | T3N1 | Ampulla | Gem + RT | R0 | Yes | Yes | Medium | 5 | Yes | 5 | Alive |
| 12 | 75 | Male | < 2 cm | 2B | T3N1 | Ampulla | None | R0 | No | No | Medium | 5 | Yes | 7 | Dead |
| 13 | 66 | Female | < 2 cm | 2B | T2N1 | Biliar duct | Gem + RT | R0 | Yes | Yes | Medium | 11 | Yes | 37 | Alive |
| 14 | 51 | Female | > 2 cm | 2B | T3N1 | Pancreas | Gem + RT | R1 | Yes | Yes | Medium | 22 | No | 22 | Alive |
| 15 | 80 | Female | < 2 cm | 2B | T3N1 | Biliar duct | None | R0 | Yes | Yes | Medium | 0 | No | 0 | Dead |
| 16 | 51 | Female | < 2 cm | 2A | T3N0 | Pancreas | Gemcitabine | R0 | Yes | Yes | Medium | 8 | Yes | 11 | Alive |
| 17 | 62 | Female | < 2 cm | 1B | T2N0 | Ampulla | None | R0 | No | No | Medium | 15 | No | 15 | Alive |
| 18 | 76 | Female | < 2 cm | 2B | T3N1 | Pancreas | None | R0 | Yes | Yes | Medium | 2 | Yes | 3 | Dead |
| 19 | 59 | Male | < 2 cm | 2B | T3N1 | Biliar duct | None | R0 | Yes | Yes | Medium | 22 | Yes | 54 | Alive |
| 20 | 74 | Female | < 2 cm | 2B | T3N1 | Ampulla | None | R0 | No | No | Medium | 14 | Yes | 51 | Alive |
| 21 | 76 | Male | < 2 cm | 1B | T2N0 | Ampulla | None | R0 | Yes | Yes | Medium | 25 | Yes | 32 | Alive |
| 22 | 82 | Male | > 2 cm | 2B | T3N1 | Biliar duct | None | R0 | Yes | Yes | Medium | 1 | Yes | 13 | Dead |
| 23 | 74 | Male | > 2 cm | 2A | T3Nx | Biliar duct | None | R0 | Yes | Yes | Medium | 6 | Yes | 8 | Alive |
| 24 | 56 | Male | < 2 cm | 2A | T3N0 | Pancreas | None | R0 | Yes | Yes | Low | 11 | Yes | 11 | Alive |
| 25 | 68 | Male | > 2 cm | 2B | T3N1 | Pancreas | None | R0 | Yes | Yes | Low | 5 | No | 5 | Alive |
| 26 | 70 | Female | > 2 cm | 2A | T3N0 | Pancreas | Gemcitabine | R0 | Yes | Yes | Low | 26 | No | 26 | Alive |
| 27 | 79 | Female | < 2 cm | 2B | T3N1 | Pancreas | None | R1 | Yes | Yes | Low | 10 | No | 10 | Alive |
| 28 | 60 | Female | < 2 cm | 2B | T3N1 | Pancreas | None | R0 | Yes | Yes | Low | 0 | No | 0 | Alive |
| 29 | 66 | Male | > 2 cm | 2B | T3N1 | Pancreas | Gemcitabine | R0 | Yes | Yes | Low | 21 | Yes | 35 | Alive |
| 30 | 57 | Female | > 2 cm | 2B | T3N1 | Biliar duct | Gemcitabine | R0 | Yes | Yes | Low | 1 | Yes | 6 | Dead |
| 31 | 78 | Female | > 2 cm | 2A | T3N0 | Ampulla | None | R0 | Yes | Yes | Negative | 9 | Yes | 11 | Alive |

**S1 Table. Clinical and pathological information of patients recruited in the study.**

cm: centimetres; Gem: Gemcitabine; RT: radiotherapy; R: resection margins; PFS: progression-free survival; OS: overall survival.
